# Associations between neurolinguistic deficits and personality traits in people with epilepsy

**DOI:** 10.3389/fneur.2024.1416713

**Published:** 2024-10-16

**Authors:** Nikitas Floros, Nikolaos Papagiannakis, Andreas Kyrozis, Elisabeth Chroni, Panagiotis Polychronopoulos

**Affiliations:** ^1^Department of Neurology, University of Patras, Patras, Greece; ^2^1st Department of Psychiatry, Eginiteion Hospital, University of Athens, Athens, Greece; ^3^1st Department of Neurology, Eginiteion Hospital, University of Athens, Athens, Greece

**Keywords:** personality, epilepsy, neuropsychiatry, neuropsychology, neurolinguistics, pragmatology

## Abstract

**Introduction:**

People with epilepsy (PWE) have been hypothesized to have higher prevalence of personality disorders and cognitive disorders. The objective of this study was to investigate the controversial notion of “epileptic personality,” a series of supposedly specific personality traits of people with epilepsy (PWE).

**Methods:**

For this purpose, 29 individuals with Mesial Temporal lobe Epilepsy (MTLE) and 23 with Juvenile myoclonic epilepsy (JME) as confirmed by electroencephalography (EEG), MRI scans and clinical examination, underwent a thorough neuropsychological and personality assessment. The resulting neuropsychological profiles were statistically analyzed considering possible personality disorders, character traits, cognitive and linguistic deviations from 20 healthy controls (HC).

**Results:**

Our findings suggest accumulative cognitive and linguistic deficits in individuals with epilepsy compared to controls. It is possible that these might be misinterpreted as personality disorders. Specifically, personality traits (*p* = 0.049) and verbal fluency (*p* = 0.013), were significantly different between PWEs and controls. Also, the type of epilepsy and lateralization seem to affect executive function (*p* = 0.049) and pragmatology scores (*p* < 0.001), exhibiting differences in subgroup analysis.

**Discussion:**

Different theories are considered as plausible pathophysiological explanations for the aforementioned differences. This research might serve as a basis to further investigate the cognitive aspects of epilepsy and possible pharmacological interventions, which are currently lacking.

## 1 Introduction

Through the 20th century psychiatric and behavioral disorders (PBDs), have been recognized as important aspects of epilepsy (1–4). Several data support that anxiety, mood, psychotic or personality disorders are common in epilepsy (5, 6). It is estimated that these conditions affect on average 30–50% of people with epilepsy (PWE), at rates two or three times higher than people without epilepsy (7).

Initially the PBDs of PWE were not attributed to underlying epileptic pathology but rather to factors such as antiepileptic drugs and psychosocial comorbidity. However, some clinicians suggested intrinsic epileptogenic biological factors contributing to a more complex correlation between PBDs and epilepsy (8, 9). During the early 21^st^ century, the concept of the “bidirectional association” between epilepsy and psychiatric comorbidity arose, based on observations of higher incidence of PBDs in PWE. Investigators realized that in some patients the psychiatric symptoms preceded the onset of seizures (10, 11). This indicated that the PBDs resulted from the same underlying pathophysiology involved in seizures themselves (12). Nowadays this concept has been widely accepted, most notably for depression (13, 14).

Among PBDs that have been linked to epilepsy, the issue of comorbid personality disorders (PD), is the most controversial. Axis II disorders in the general population range between 6 and 13%, whereas in PWEs between 4 and 35% and several epidemiological studies report higher rates in comparison to other neurological disorders (15). For the diagnosis of personality disorder, DSM-5 criteria are based on (1) cognition, including narrative and thinking form in an unconstrained context (especially abstract reasoning), (2) emotional integrity and (3) relational patterns (16). However, diagnosis remains elusive, firstly due to significant overlap between different psychiatric disorders and secondly due to poor specificity of psychometric questionaries (46% overlap in patients with personality disorders) (17). Furthermore, there is conflicting scientific evidence concerning the impact of epilepsy in personality. Some researchers adopted the term “epileptic personality,” to describe the higher incidence of cluster C, a subtype of PDs in PWEs. They also observed specific traits such as “circumstantiality” which describes the habit of contextually inappropriate speech (18). Nevertheless, findings remain inconsistent and further research is required (19, 20).

Most studies have suggested that PD commonly occurs in Temporal Lobe Epilepsy (TLE) a type of focal epilepsy and also in Juvenile Myoclonic Epilepsy (JME), a type of generalized epilepsy (21). However, some personality traits might emerge as part of more complex PBDs in PWE as a result of ictal activity. For instance, people with TLE can exhibit a specific interictal behavioral syndrome the “Gastaut–Geschwind syndrome” (9), which consists of alterations in sexual behavior, irritability, increased religiosity, hypergraphia and circumstantiality. This syndrome has been attributed to limbic system dysfunction in individuals with TLE. Impairment of conversational language is often described as verbosity, which is attributed to dysfunctional cognitive linguistic networks manifesting as tangentiality, longer speaking time, longer duration of pauses, higher proportion of repetitive or redundant statements, less attention to prosody and facial expressions (22). Disrupted projections from temporal to frontal lobe regions in TLE may play a pivotal role in narrative discourse and informational integration (23, 24). However, the existence of this phenomenon remains controversial, since only few studies have been conducted using valid diagnostic tests and specific criteria have yet to be developed for this condition.

On the other hand, with regards to some people with JME, many clinicians have observed “novelty seeking behaviors” often linked with poor treatment compliance (3). Although these may be inherently common in adolescent behavior, they may also be related to epilepsy (25). Neuropsychological testing and advanced imaging techniques in individuals with JME suggest dysfunction of networks linking motor and cognitive neuronal centers (21).

The need to address the nature of personality traits in PWEs was the starting point of this research. The aim was to identify the relationship between atypical behaviors observed in PWEs and personality disorders, and their connection to linguistic and cognitive deficits in skills necessary for social interaction. It is hypothesized that a thorough examination can reveal component defects in specific cognitive tasks, which in aggregate might be misinterpreted as psychiatric symptoms in PWEs (26).

## 2 Materials and methods

### 2.1 Participants and demographics

This study included 72 participants, 23 individuals with JME (*n* = 23, Group 1) and 29 with MTLE (*n* = 29, Group 2) which were recruited from the epilepsy outpatient department of the Universities of Patras and Athens. Additionally, 20 healthy controls (*n* = 20, Group 3), with matched sociodemographic profiles were enrolled from hospital staff via convenience sampling, with no previous history of epilepsy psychiatric disorders or other chronic diseases.

Sociodemographic and clinical data on age, sex, level of education, age of onset of epilepsy, duration of illness, type of seizure, family history of epilepsy, EEG and MRI findings, side of lesion in patients with MTLE and antiepileptic treatment in patients were gathered (Table 1).

**Table 1:** Demographics.

		Individuals					
		TLE (HS)		JME		control	
		Median	Count	Median	Count	Median	Count
Age		37 (24.8–46.5)		22 (18–26)		29.5 (21.5–39)	
Sex	Male		13		7		9
	Female		16		16		11
Education (years)		14.5 (12–16)		13 (12–14.5)		16 (12–17)	
Age of onset of epilepsy		16 (13–24)		16 (14.8–19)		.	
Duration of epilepsy (years)		22.5 (12.8–29)		4.5 (3–6.25)		.	
MRI	No findings		8		23		0
	Right		6		0		0
	Left		9		0		0
EEG	No findings		0		0		0
	Right		9		0		0
	Left		15		0		0
	Generalized		0		23		0
Medication	Mono		6		16		0
	Poly		21		4		0
Drug resistance	Yes		18		4		0
	No		9		16		0

The study was approved by the University of Patras Ethics Committee, in accordance with ethical standards of the 1964 Declaration of Helsinki. All participants provided written informed consent. Clinical examination took place in the neurological department of Patras University Hospital and Eginiteion University Hospital, Athens, Greece. Neuropsychological evaluations were conducted by a single trained psychiatrist.

Diagnosis of JME and MTLE were based on electroclinical findings and consensus criteria according to the International League Against Epilepsy (ILAE) classification (27–29). Seizure activity was measured using routine EEG or video EEG monitoring. All patients with MTLE also had clear MRI findings consistent with unilateral or bilateral Mesial Temporal Sclerosis (MTS) and concordant interictal or ictal EEG findings. None of them had undergone amygdalohippocampectomy, or received Topiramate, Zonisamide which is associated with cognitive decline (30). Inclusion and exclusion criteria are listed on Table 2.

**Table 2:** Participation criteria.

Inclusion criteria	Exclusion criteria
Age of onset of myoclonus between 10 and 25 years.	Any evidence of symptomatic or progressive myoclonic epilepsy.
Normal intelligence.	Severe depressive symptoms based on BDI classification.
EEG shows a normal background and interictal generalized spikes and/or poly-spikes and waves.	EEG showing predominant focal interictal epileptiform discharges or abnormal background.
Myoclonic Jerks predominantly occurring on awaking.	Unwilling or unable to provide informed consent for the study.
Greek language.	Current major psychiatric episode.
	History of developmental or other neurological condition.
	Treatment with Τopiramate or Ζonisamide.

This study used structured questionnaires to quantify specific aspects of cognition and behavior (7) in two distinct types of epilepsy Mesial Temporal Lobe Epilepsy (MTLE), JME and controls. The resulting personality and cognitive profiles along with EEG, MRI and other clinical data (such as drug resistance, family history, medication, age of onset, duration of epilepsy) were compared between the two types of epilepsy and also between PWEs vs. healthy controls. These comparisons may unveil certain cognitive deficits, providing new evidence about the contextual use of language and the supposed personality pathology of PWEs.

### 2.2 Overview of neuropsychological instruments and discourse elicitation

This study used a standardized personality questionnaire (Personality Dimensions Questionnaire, PDQ4) that categorizes participants on a continuum of personality styles and then estimates the severity in different items, e.g., social functioning. The test includes a corresponding interview, where participants elaborate on the hardships that have been highlighted in the above items in everyday situations, indicating a possible personality disorder diagnosis. This questionnaire also yields a composite score of total functioning. With this procedure character traits were quantified. Also, a Pragmatic Rating scale (PRS), which assesses prosody and contextual language was used during this interview to quantify parameters of spontaneous speech such as speaking time, duration of pauses, vocal tone, topic shifts and facial expressions. These measurements were used to compare differences between PWEs and controls. Also, semantic and verbal fluency tests were administrated as part of a basic cognitive assessment, which included speed processing (Trail making tests, TMT), attention (STROOP), spatial and verbal memory modalities (Benton Visual Retention Test, Rey Auditory Verbal Learning Tests, RAVLT). The selected cognitive tests gave emphasis to core prefrontal cognitive skills and linguistic modalities. All test administrations adhered to standardized procedures. Using the above tools, both verbal and non-verbal interaction patterns along with cognitive and emotional characteristics were evaluated in both types of PWEs and controls and the outcome was compared to investigate differences between the groups.

**Figure 1:**
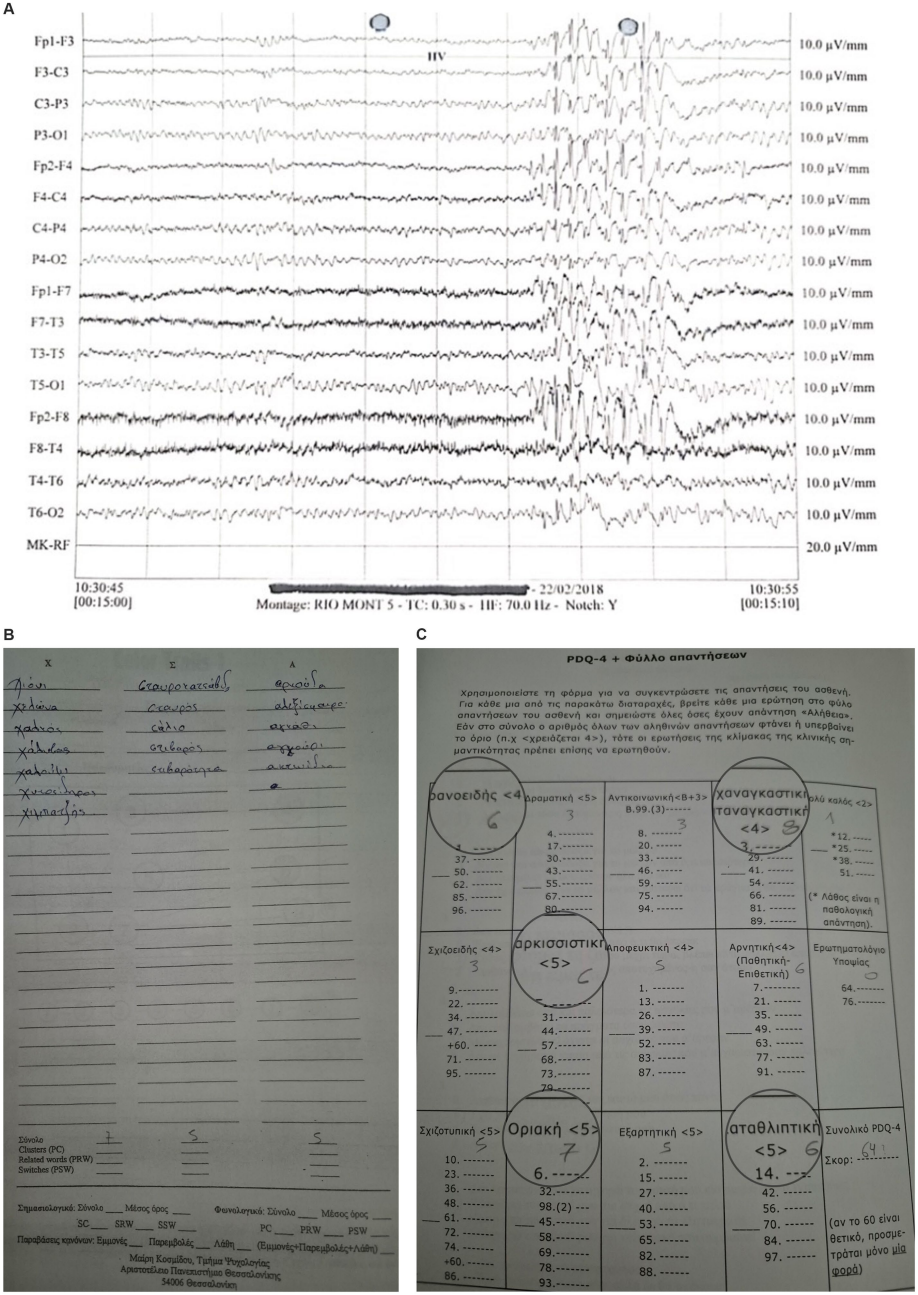
JME Case-EEG: Bilateral and synchronous generalized spike/polyspike/slow wave discharges (4-6Hz) over a normal background, exhibiting borderline personality traits and poor phonological verbal fluency.

**Figure 2:**
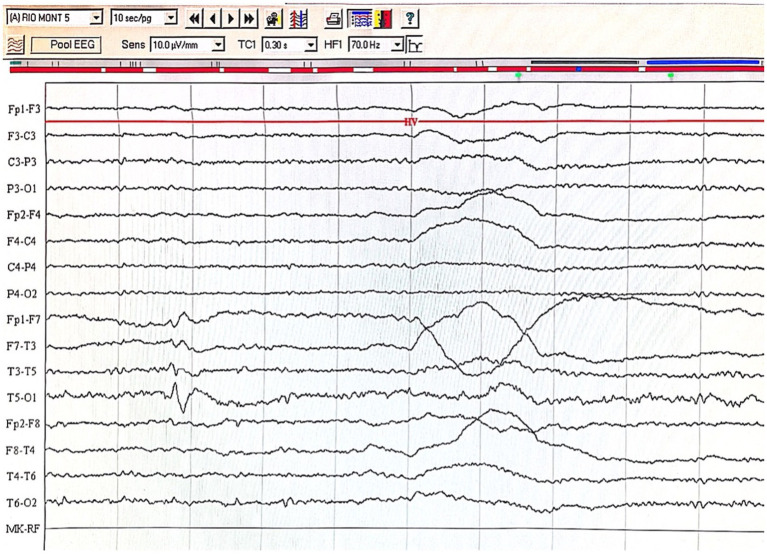
An individual with MTLE exhibiting single spike and slow wave focal discharge in the left temporal area with phase reversing (T5). Personality testing revealed cluster B and C traits while neuropsychological examination revealed difficulties in the Benton visual retention test.

**Figure 3:**
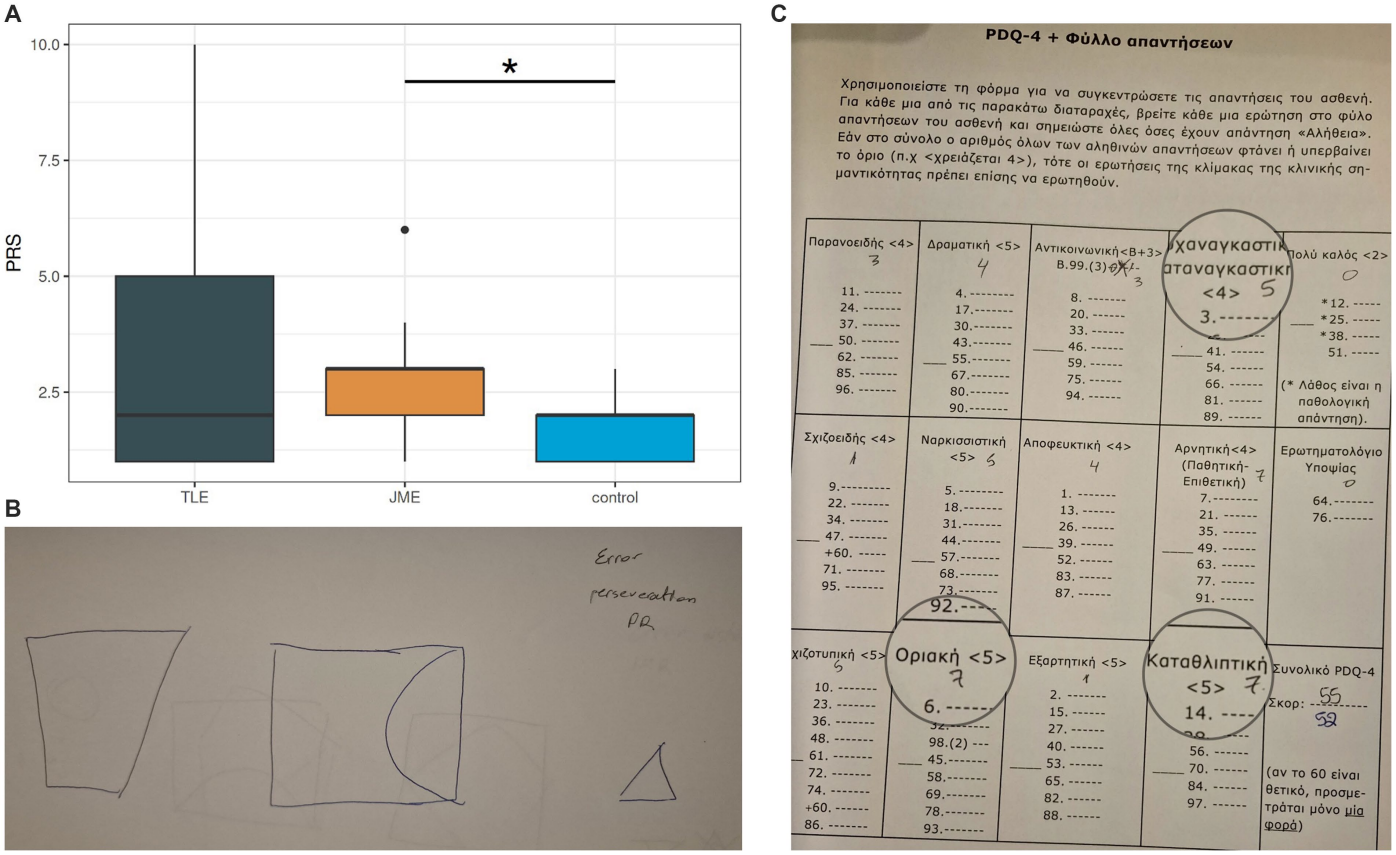
Pragmatic scores among groups Note. PRS = pragmatics scale score, higher scores = more pragmatology hardships, TLE = patients with Temporal Lobe Epilepsy, JME = patients with Juvenile Myoclonic Epilepsy.

**Figure 4:**
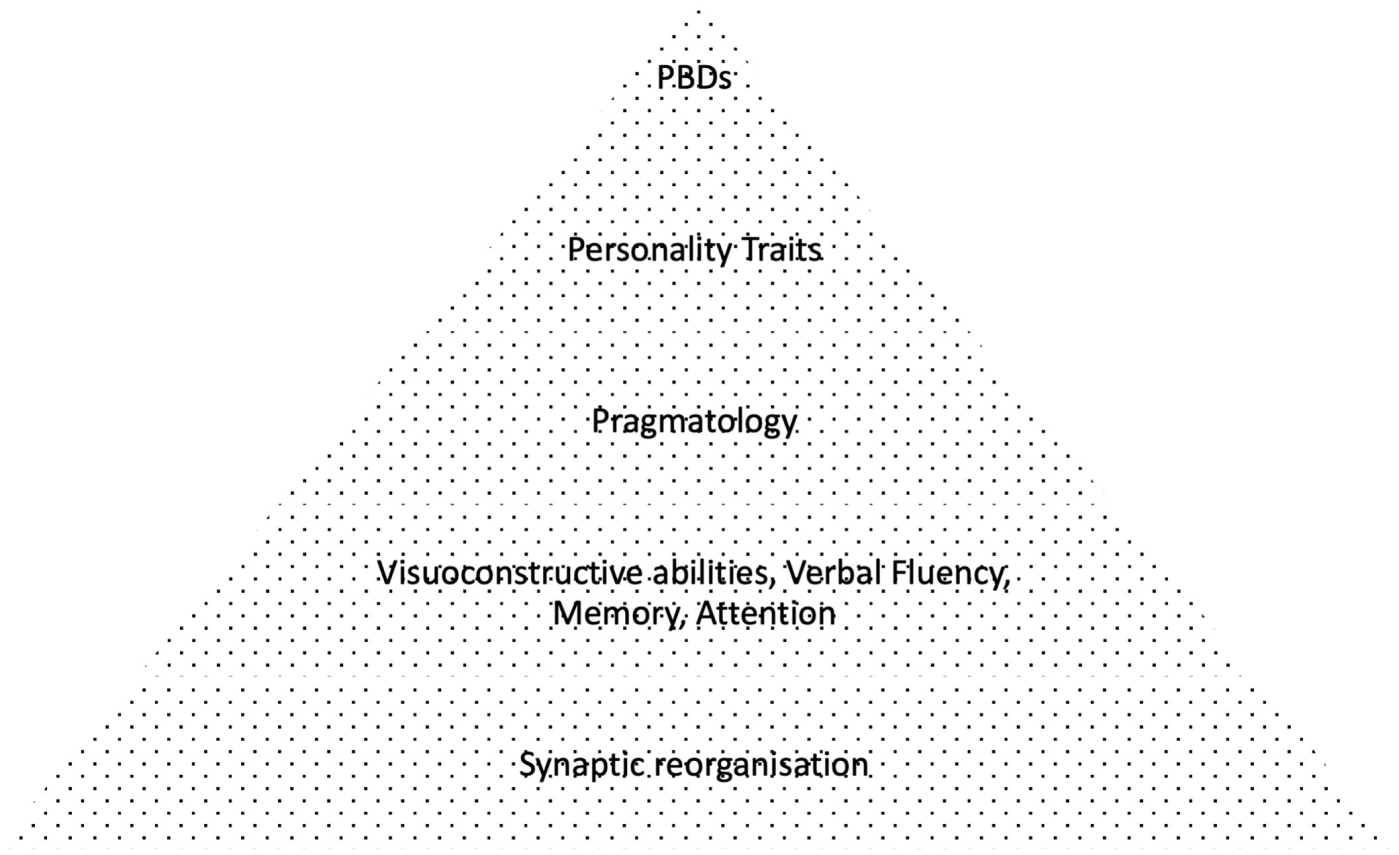
Complex mental symptoms based on component defects as conceptualized in the study.

1. PDQ4 Questionnaire: The PDQ4 questionnaire was used to identify emotional distress and personality traits. The personality style with the highest score on this questionnaire was identified and marked. In cases where two or more personality types had similar scores, the cluster with the most points was marked, creating a continuum (31). Participants were then further interviewed based on these results to assess the impact of these traits in relationships and everyday functioning, so as to diagnose a personality disorder. Regardless of the diagnosis, the total “composite” score of each patient was measured in order to assess the level of total psychological distress (32).
2. Rey auditory verbal learning test (RAVLT): The RAVLT was conducted without a time limit. Patients were prompted if more than 10 seconds passed without disclosing completion. Scores were measured to assess hippocampal verbal memory consolidation (33).
3. STROOP test: The STROOP was used to assess prefrontal attentive and anterior cingulate cortex functions. The STROOP was administered without time limitations, with participants instructed to “take it slow” to minimize attentional load. The total number of errors was measured. This subtest was of particular interest due to the potential difficulties patients might have in adapting their attention to external stimuli. The STROOP test captured the potential impact of attentional anticipation, adaptation and planning in contextual deficits (34).
4. Trail making test A&B (TMT): Participants were given the same labyrinth problem (after having been trained on an example) and were urged to connect the dots as quickly as possible. The total time was measured. The TMT addresses frontal lobe processing speed in problem solving and top-down suppression skills (35).
5. Benton visual retention test (BVRT): The BVRT was used to assess visuocontsructive abilities located in anatomically different brain regions. Stimuli were shown to the participants for 10 seconds, after which they were asked to draw the pattern from memory. The total number of errors was measured. Qualitative data were collected but were not included in this study (36).
6. A Pragmatics Rating Scale (PRS): The PRS was used to assess prosody and contextual language use during the examination. The actual number of semantic shifts in spontaneous speech was recorded along with other clinical details including vocal tone, narrative time, facial expressions, gestures, emotional concordance. At the end of the examination a total score was measured for each participant (37, 38).
7. Beck Depression Inventory for medical patients (BDI): The BDI was used to identify depressive symptoms. Since depression is known to affect cognitive functioning, participants with severe symptoms were excluded from the analysis (score > 10/21) (39).
8. Verbal fluency tests (fluency): Verbal fluency encompassing both semantic and phonological aspects, were used to assess ventral and dorsal auditory stream functioning. Specifically, the auditory dorsal stream (ADS) was clinically assessed by measuring the phonological lexicon. According to theory, the ADS monitors the semantic shift of discourse, by keeping track of the perceived vs. the emitted speech. These measurements were invaluable for further statistical comparison between fluency and pragmatics scale scores to assess the integrity of linguistic function in PWEs (40). Similar comparisons were made for auditory ventral stream (AVS) function, as measured through semantic lexicon scores, to investigate potential correlations with the phonological and pragmatics scores of PWEs (41, 42) (Figures 1–4).

### 2.3 Data analysis and targets

Statistical analysis was performed using R version 4.1 with RStudio and STATA. All data underwent a normality test (Shapiro–Wilk) and were found to be non-normally distributed. The significance of the presence of differences between continuous measurements in the different groups was assessed with Kruskal-Wallis’s test, with Dunn’s *post hoc* test. Chi square test was used to assess associations between categorical variables. Spearman’s correlation coefficient was used to assess the correlations between the results of the two different continuous variables. Patient and control groups were matched for age, gender, education and demographics. For tables we used the SPSS statistical package. We compared the three groups across the PDQ4 (character traits), PRS (pragmatics), Verbal fluency, STROOP (executive), BDI (Depression), RAVLT (Memory), BVRT (Visuospatial) and Lateralization domains. Data presented as median (interquartile range). Each clinical group was compared with controls, and JME versus MTLE groups were studied in order to verify differences in the aforementioned cognitive functions between these groups. Correlations with clinical parameters such as the side of the lesion in patients with MTLE, duration of epilepsy, seizure frequency, family history and the interrelations with the above deficits were examined. Statistical significance as denoted by *p* values was calculated to measure the strength of these associations. The Bonferonni-Hochman false discovery rate correction was applied to account for multiple comparisons.

## 3 Results

### 3.1 Participant characteristics

PWEs and healthy controls were comparable across most demographic and neuropsychological characteristics providing a sound foundation to compare cognitive and personality aspects (Table 1).

### 3.2 Group comparisons and correlations

According to our findings, PWEs varied significantly compared to controls, in verbal fluency (*p* = 0.013) and personality traits as exhibited by differences in total PDQ4 scores (*p* = 0.005), in spite of a personality disorder diagnosis. Executive function (*p* = 0.069), visuospatial memory (*p* = 0.094) and pragmatic language skills (*p* = 0.095) exhibited a statistical tendency that marginally did not reach significance with 0.05 as confidence interval. In the omnibus comparison of the three groups, personality (PDQ4 *p* = 0.035), executive function (STROOP *p* = 0.011, TMT *p* = 0.011) verbal memory (RAVLT list A *p* = 0.013), pragmatology (PRS *p* = 0.007) and phonological fluency (fluency phono *p* = 0.001) also had significant differences.

In post-hoc analyses, when compared to controls, individuals with JME underperformed in executive function (STROOP, *p* = 0.049) and pragmatics (PRS, *p* < 0.001, while individuals with MTLE exhibited important differences in personality traits (PDQ4, *p* = 0.021) and a statistical tendency in verbal fluency (*p* = 0.078). Amongst individuals with MTLE and different lesion sites, lateralization was associated with differences in executive function (*p* = 0.02) and a statistical tendency with verbal memory (*p* = 0.098), with left lesions having a greater impact than right ones.

**Figure 5:**
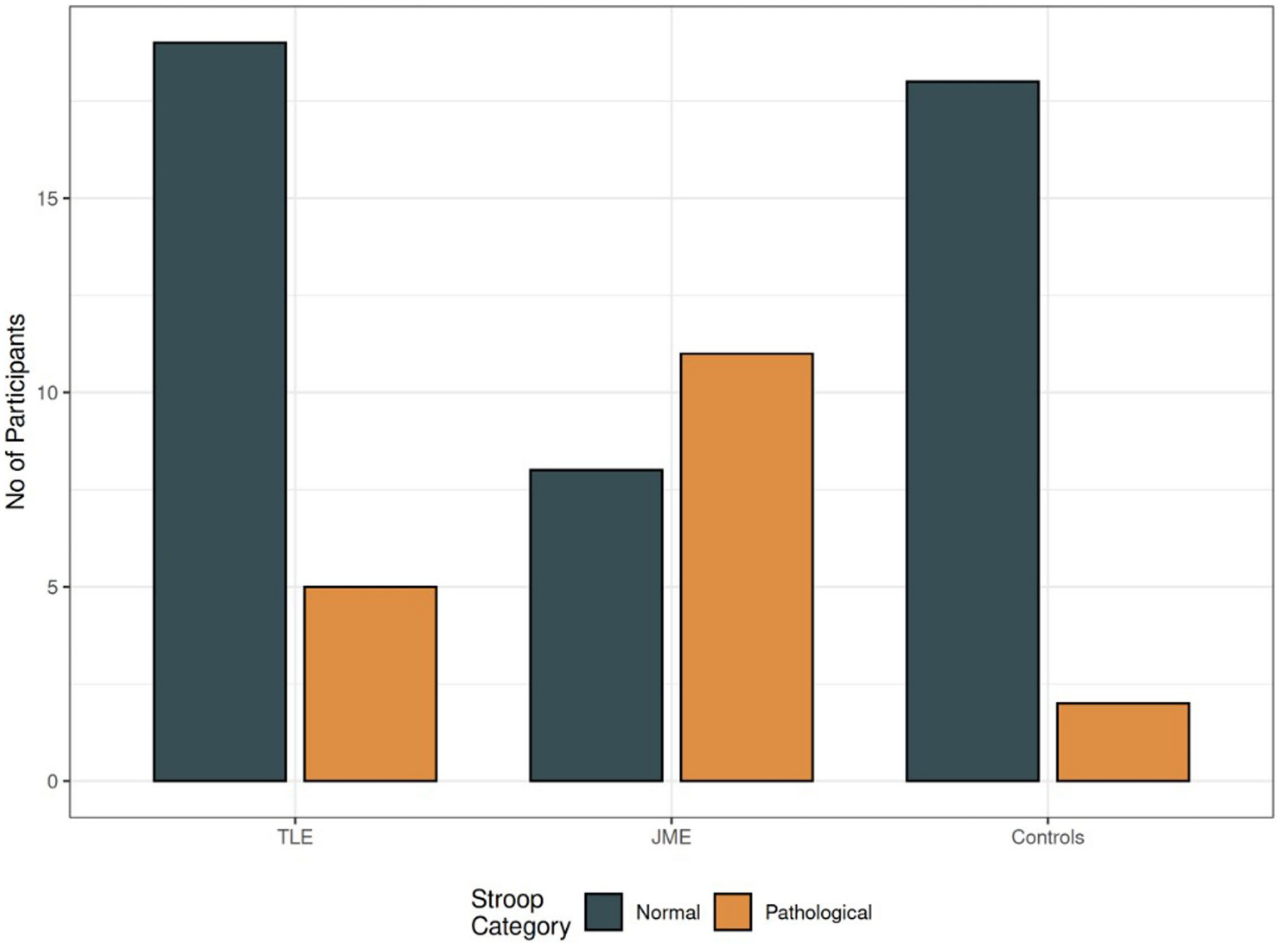
STROOP differences amongst groups. Note. Grey: number of individuals with normal STROOP test, Orange: number of individuals with errors, TLE = patients with Temporal Lobe Epilepsy, JME = patients with Juvenile Myoclonic Epilepsy.

Amongst PWEs, individuals with MTLE had more elevated verbal memory skills than individuals with JME although it did not reach statistical significance (*p* = 0.095). Also, individuals with JME exhibited more frequent STROOP pathology in comparison with MTLE and controls, using logistic regression (Figure 5).

**Figure 6:**
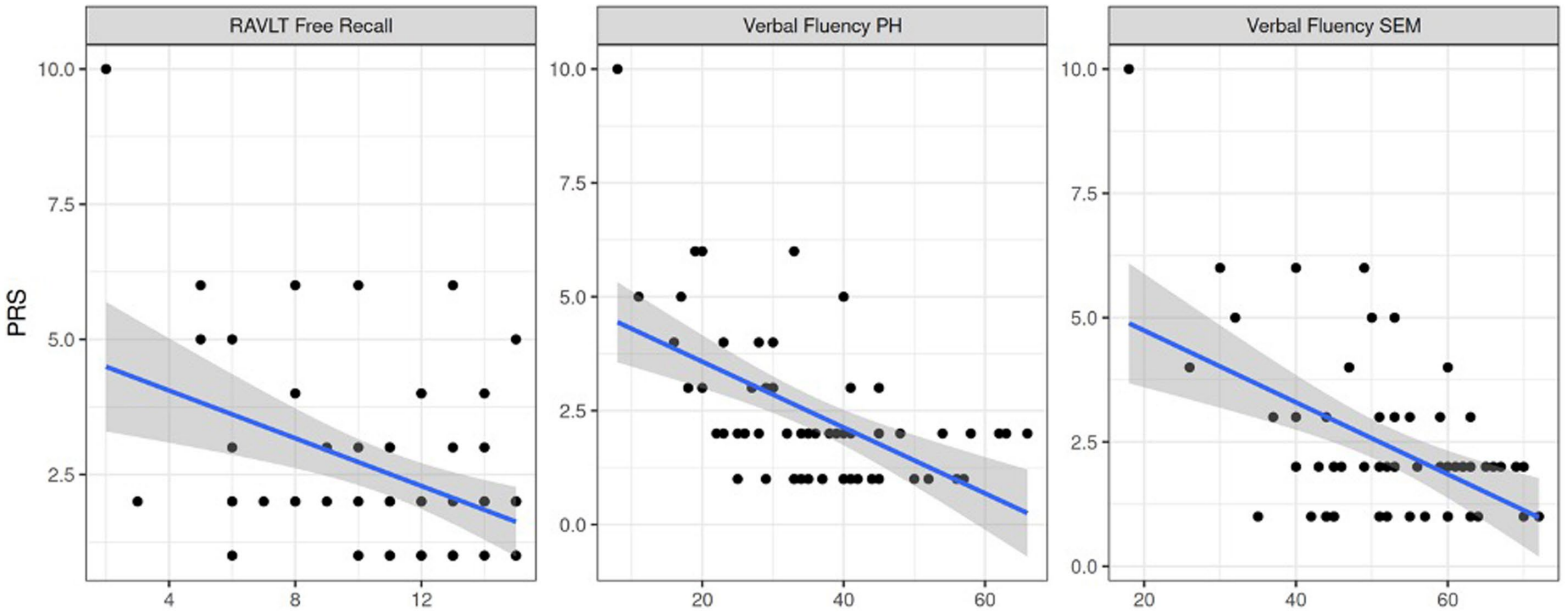
Pragmatics association to verbal memory retention, phonological and semantic verbal fluency respectively. Note. PRS = pragmatic scale score, RAVLT Free Recall = RAVLT free recall task score, Verbal Fluency PH = phonological verbal fluency score, Verbal Fluency SEM = semantic verbal fluency score.

Circumstantiality in both PWEs and controls was statistically associated with poorer verbal fluency (semantic and phonological, *p* = 0.04) and verbal memory (*p* < 0.001, Figure 6). Delays and poor coherence were among the most common pragmatologic deficits. These associations between RAVLT, PDQ4 and PRS scores might explain character traits as part of linguistic deficits in PWEs.

Finally, PWEs exhibit higher rates of depressive symptomatology than healthy controls (*p* = 0.02). Higher BDI scores were systematically related to poorer overall neuropsychological performance in our assessments, which confirms the well-established link between depression and poor cognitive function. Statistical correlations that arose between the above factors and *p*-value scores are included in Table 3. All correlations between tests are mentioned in Table 4.

**Table 3:** Associations between different groups.

Test	Patient group	PWEs vs. control	JME vs. control	MTLE vs. control	MTLE RIGHT vs. control	MTLE LEFT vs. control
Personality (PDQ4)		**0.017 (0.049)**	*0.053 (0.092)*	**0.004 (0.021)**	*0.08 (0.094)*	
Pragmatics (PRS)		*0.065 (0.095)*	**<0.001 (<0.001)**			
Executive (Stroop)		**0.031 (0.069)**	**0.015 (0.049)**			
					**0.004(0.02)** RIGHT compared to LEFT	
Fluency		**0.001 (0.013)**	**0.012 (0.049)**	**0.042 (0.078)**		**0.015(0.048)** (sem)/**0.078**(0.095) (pho)
Executive (TMT)		**0.039 (0.078)**	**0.021 (0.054)**	*0.098*		**0.032(0.069)** (1)
Verbal memory (RAVLT)			*0.094 (0.097)*	*0.085* (free) (0.096)		*0.071* (free) (0.094)
			*0.078(0.095)* JME compared to MTLE		*0.093* (0.098) List A RIGHT compared to LEFT	
Spatial memory (BVRT)		*0.08 (0.094)*		*0.064 (0.094)*		**0.002 (0.017)**

The main *p*-values are the result of Kruskal-Wallis test. *p*-values in brackets are after applying the BH correction. Bold: significant (*p* < 0.05), Italics: marginal, sem. = semantical score, pho. = phonological score, 1 = TMT1 score, free = RAVLT free recall task score, list A = RAVLT list A score.

**Table 4:** Correlation between neuropsychological tests.

Var1	Var2	rho	P-value	Adjusted p-value
PDQ4	STROOP	0,136	0,295	0,403
	PRS	0,01	0,94	0,958
	**BDI**	0,502	0	0
	TMT1	0,279	0,028	0,06
	TMT2	0,197	0,124	0,214
	ver.fl.sem	−0,286	0,024	0,054
	ver.fl.phono	−0,173	0,179	0,272
	RAVLT listA	−0,085	0,509	0,615
	RAVLT listB	−0,105	0,411	0,52
	RAVLT Free Recall	−0,023	0,856	0,916
	BENTON errors	0,132	0,301	0,405
STROOP	PRS	0,056	0,673	0,76
	BDI	−0,061	0,64	0,738
	TMT1	0,185	0,157	0,242
	TMT2	0,186	0,155	0,242
	ver.fl.sem	−0,165	0,209	0,3
	ver.fl.phono	−0,29	0,025	0,054
	RAVLT listA	−0,097	0,457	0,557
	RAVLT listB	−0,019	0,887	0,916
	RAVLT Free Recall	0,021	0,874	0,916
	BENTON errors	−0,08	0,541	0,639
PRS	BDI	0,061	0,639	0,738
	TMT1	0,23	0,077	0,148
	**TMT2**	0,419	0,001	0,005
	ver.fl.sem	−0,252	0,052	0,103
	**ver.fl.phono**	−0,521	0	0
	**RAVLT listA**	−0,449	0	0,002
	**RAVLT listB**	−0,413	0,001	0,005
	RAVLT Free Recall	−0,28	0,029	0,06
	**BENTON errors**	0,352	0,005	0,018
BDI	TMT1	0,201	0,118	0,213
	TMT2	0,188	0,144	0,237
	ver.fl.sem	−0,165	0,201	0,293
	ver.fl.phono	−0,018	0,89	0,916
	RALVT listA	−0,197	0,122	0,214
	RAVLT listB	−0,259	0,04	0,081
	RAVLT Free Recall	−0,114	0,374	0,478
	BENTON errors	0,208	0,103	0,192
TMT1	**TMT2**	0,667	0	0
	**ver.fl.sem**	−0,296	0,02	0,047
	**ver.fl.phono**	−0,383	0,002	0,008
	**RAVLT listA**	−0,338	0,007	0,023
	**RAVLT listB**	−0,293	0,021	0,048
	RAVLT Free Recall	−0,193	0,134	0,226
	**BENTON errors**	0,438	0	0,003
TMT2	**ver.fl.sem**	−0,477	0	0,001
	**ver.fl.phono**	−0,513	0	0
	**RAVLT listA**	−0,498	0	0
	**RAVLT listB**	−0,369	0,003	0,012
	**RAVLT Free Recall**	−0,423	0,001	0,003
	**BENTON errors**	0,299	0,018	0,044
Fluency SEM	**ver.fl.phono**	0,632	0	0
	**RAVLT listA**	0,396	0,001	0,006
	**RAVLT listB**	0,303	0,016	0,041
	**RAVLT Free Recall**	0,355	0,005	0,016
	**BENTON errors**	−0,315	0,013	0,035
Fluency PHO	**RAVLT listA**	0,538	0	0
	**RAVLT listB**	0,451	0	0,002
	**RAVLT Free Recall**	0,403	0,001	0,005
	BENTON errors	−0,289	0,023	0,052
RAVLT listA	**RAVLT listB**	0,457	0	0,001
	**RAVLT Free Recall**	0,828	0	0
	**BENTON errors**	−0,48	0	0,001
RAVLT listB	**RAVLT Free Recall**	0,385	0,002	0,007
	**BENTON errors**	−0,364	0,003	0,012
RAVLT Free Recall	**BENTON errors**	−0,424	0,001	0,003

Correlations are noted with bold.

## 4 Discussion

Considering our findings, the primary purpose of this study was to investigate the incidence of personality disorders in PWEs compared to healthy controls, in association with secondary cognitive and pragmatologic deficits (2). It was anticipated that these cognitive component defects would be correlated in a bottom-up manner with the severity of epilepsy, as denoted by clinical parameters including drug resistance, EEG and MRI findings, family history. It was expected that lower order cognitive deficits would be impacted first (e.g., microlinguistic function, visuospatial abilities etc.), leading consequently to higher order (macrolinguistic, attention and pragmatologic) deficits and finally resulting in personality distress (Figure 4), but such direct hierarchical correlations were not observed in our findings. Nevertheless, our findings suggest a potential diagnostic overshadowing effect of excessive personality diagnosis in PWE, stemming from underlying cognitive deficits, mainly affecting executive function, verbal fluency and pragmatics, in accordance with our initial hypothesis.

Commenting on the scope of neuropsychological testing, we hypothesized that spontaneously adjusting the use of language under specific contextual conditions exerts a great stress on prefrontal domains due to high planning demands. This applies for both verbal and non-verbal communication. Such dynamic linguistic processes rely on attention, working memory and fluency, which are normally integrated into informative and coherent speech. These higher order cognitive processes could be impacted from background word retrieval deficits. To investigate that, the expressive phonological and semantic components of speech were clinically assessed using phonological and semantical fluency testing, which according to theory are controlled by two anatomically distinct networks, the auditory dorsal and ventral stream (ADS/AVS) respectively (42). These networks could be affected from reorganization processes due to ictal activity (43). Attention and adaptation strategies were assessed using the STROOP. These measurements were correlated with the ability to monitor semantic shifts in conversation during examination using pragmatic scale scores (PRS), in order to identify underlying cognitive deficits in PWEs compared to healthy controls.

Regarding the differences in group comparisons, it was shown that individuals with epilepsy and especially JME, are more likely to exhibit verbosity when output is spontaneous and unconstrained, presumably due to the impact of lexical retrieval and executive deficits, which maims adaptability within an unknown semantic space. They tend to deviate more from the topic of conversation, being less concise. In order to maintain social contact and be understood they become repetitive and loquacious. These strategies, which plausibly represent compensations for lexical retrieval deficits, might be clinically misinterpreted as personality pathology. The present findings align with neuroscientific literature on cognition and linguistics, where functionality is impacted not only during seizures but also from the resulting “synaptic reorganization” of the neural circuitry due to chronic subthreshold ictal activity (43). However, it remains unanswered why this was not marked in MTLE where the lesion cites are anatomically specific. One reason for this might be that executive function is crucial in spontaneous speech. MTLE vs. JME both had fluency issues but differed in executive abilities. This might imply that JME has a toll on frontal regions. Their verbosity aligns with the clinicians anecdotal reporting of personality changes in epilepsy, which often do not meet personality disorder criteria. TLE on the other hand seemed to have more personality traits. Further research is crucial to fully characterize the nuances of these neurolinguistic impairments.

Moreover, it was expected that severe epilepsy pathology as indicated by drug resistance, brain imaging (MRI), EEG and neurophysiological testing, would directly correlate to poorer cognitive outcomes and personality pathology, but no such correlation arose. Higher STROOP pathology was found in some participants with low pragmatics capabilities, irrespectively of “hard” evidence of damage in brain tissue.

May be the wiring of different functions in the brain is topologically chaotic. This is disproved by EEG research reflecting on pre attentive cognitive operations, involved in involuntary attention of deviant stimuli (44). Multiple networks in our brain interact to simultaneously monitor both incoming stimuli from our surroundings, along with our conscious responses to them (namely motor and mental reactions). Most of these processes take place intrinsically, at a subconscious level. In this procedure the prefrontal network and anterior cingulate gyrus modulate anticipation (prognosis) focusing our attention to different parameters of our surroundings including discussants. This organizes broader narrative strategies depending upon working memory and lexical retrieval (45, 46) to serve a particular goal or plan. This complexity might not yet be captured by modern diagnostic technology.

### 4.1 Theoretical pathophysiological background and future targets

It is already known that depression has a bidirectional association with epilepsy and it might explain some of our neuropsychological findings, even some personality traits in patients with epilepsy, as a means of expressing suffering. However, that does not explain why other chronically ill patients do not develop these specific traits. The effect of epilepsy in the remodeling of the brain and cognition might be increasing this burden.

Research has shown a huge overlap between the incidence of neurodevelopmental disorders (for example AS and ADHD) and several neuropsychiatric illnesses such as epilepsy and personality disorders (47, 48). Epilepsy is speculated to be itself developmental (known through the well-established 2hit hypothesis). It would be interesting to see if further research concurs with the results of the present study, claiming that these three clinical entities (Pragmatologic disorder, Epilepsy and Personality disorder) are linked through neurolinguistic traits. One plausible explanation might be that they share a common pathogenetic background, although that seems rather oversimplistic. A more refined explanation might assume the existence of different pathogenetic mechanics that share a common feature of disrupting lingual modalities, such as symbol formation and mentalization capabilities, which according to Fonagy et al. (49), emerge in the early years of the developing brain (type III, 50). Speculatively, that would lead to character traits such as poor understanding of contextual affective information and poorer emotional representations, less social interaction, altered limbic and prefrontal functioning, resulting in maladaptive behaviors. These traits largely coincide with various theories regarding the pathophysiological substrates of personality by former theorists Eysenck (50), Gray, Zuckerman (51), Cloninger (52), and possibly reflect poor “mentalization” capacity (49, 53). All these theories are criticized because personality is regarded a profoundly intricate epiphenomenon, influenced by numerous sociocultural predisposing and precipitating factors, that often cannot be reduced to discrete traits or underlying brain loci.

The study of Event Related Potentials in several neurological and psychiatric diseases has yielded interesting results in adaptive attention, monitoring internal and external auditory stimuli (44). Future research could incorporate these advanced EEG techniques for the investigation of specific ERPs related to pragmatics and further linguistic deficits in PWEs. Interestingly, certain ERPs such as Miss Match Negativity and P3a have been coupled with NMDA receptors dysfunction, which might be a suitable target for pharmacological interventions and are already undergoing extensive research in order to support cognitive function in schizophrenia (54).

Finally, an important issue from our everyday clinical experience with PWEs is stigmatization. Patients are frequently treated differently, even prior to their examination, especially in Emergency Rooms, based on their medical history and socioeconomic status. Our concentrated efforts must be to eliminate bias regarding both diagnostic, research and therapeutic interventions in our patients. The medical community should adapt slowly and rationally to the many complexities of human nature. The scientific fragmentation of human behavior and cognition must not stop us from responding to our patients’ multiple needs as a whole.

## 5 Limitations

Regarding the limitations of the presented study, the number of participants remains small. Since all our patients received treatment, bigger studies can examine the effect of anticonvulsive medication on cognition, although drugs that are already known to affect cognition and language (such as Topiramate and Zonisamide) were excluded from this study. Also, some tests were used in an experimental manner. Widely accepted tools must be implemented by the neuropsychiatric community. Due to COVID 19 lockdown conditions more assessments were discontinued, but future research might have an opportunity to gather larger samples and recruit more evenly distributed groups to confirm the veracity of our findings.

## 6 Conclusion

There is good evidence that more emphasis must be given to cognitive, linguistic and personality traits in PWEs. It is suggested that cognitive assessment focusing on executive function and verbal fluency, might convey insight in PWEs that present personality issues after other major psychiatric comorbidities like depression are excluded. Future EEG studies could further investigate ERPs related to specific cognitive tasks, with a view to more refined diagnostic tools and strengthening our medicinal arsenal. Perhaps research could also shed further insight on the association of neurodevelopment and cognitive deficiencies implicated in epilepsy. Until then we urge against the stigmatization of PWEs.

## Data Availability

The raw data supporting the conclusions of this article will be made available by the authors, without undue reservation.
